# Cellular and molecular events of inflammation induced transdifferentiation (EMT) and regeneration (MET) in mesenteric mesothelial cells

**DOI:** 10.1007/s00011-020-01400-7

**Published:** 2020-09-12

**Authors:** Viktória Zsiros, Anna L. Kiss

**Affiliations:** Department of Anatomy, Histology and Embryology, Semmelweis University, Tűzoltó u. 58, Budapest, 1094 Hungary

**Keywords:** Mesenteric mesothelial cells, Inflammation, Epithelial-to-mesenchymal transition, Mesenchymal-to-epithelial transition, Regeneration

## Abstract

In this review we summarize the cellular and molecular events of inflammation induced epithelial-to-mesenchymal (EMT) and mesothelial-to-macrophage transition (MET) during regeneration. Since the receptor transmits the environmental stimulus, downregulating or upregulating the process on an epigenetic level, the intracellular localization of receptors (signaling organelles: early endosomes or lysosomal degradation: late endosomes) plays a crucial role in the signaling events regulating inflammation and regeneration. Therefore, we focused on the internalization of the receptors as well as the intracellular compartmentalization of signaling molecules during EMT and MET. The review draws the reader’s attention to the plasticity of mesothelial cells and supports the idea that during inflammation an ambient macrophage population might derive from mesothelial cells.

## Introduction

Mesothelial cells covering the surface of the mesentery are simple squamous cells originating from the embryonic splanchnopleura. Under inflammatory stimuli these cells undergo a series of morphological and biochemical changes during which they lose their epithelial character, and transdifferentiate into mesenchymal cells [1]. They become cuboidal in shape, the volume of the cytoplasm and the number of the cytoplasmic organelles (rough ER, mitochondria, and Golgi vesicles) are dramatically increasing. They lose their polarity, the cell junctions are disassembling by E-cadherin and ß-catenin down-regulation [2, 3], the basement membrane is degrading [4], and the cytoskeleton becomes rearranged. The mesothelial cells start to express mesenchymal markers (*N*-cadherin, vimentin, α-smooth muscle actin) and become highly mobile cells. Besides expressing mesenchymal markers, epithelial cells acquire the ability to produce extracellular matrix components, inflammatory cytokines, fibrogenic and angiogenic factors [5].

All these morphological and biochemical changes are characteristic of epithelial-to-mesenchymal transition (EMT). EMT is an important, reversible biological process, when polarized epithelial cells undergo a complex proteomic remodeling to assume mesenchymal characteristics [6]. Three types of EMT have been distinguished so far: type I EMT during embryogenesis; type II EMT associated with inflammation, wound healing, tissue regeneration and organ fibrosis and type III occurring during tumorigenesis [7]. EMT is triggered by various extracellular signals including cytokines, growth factors, and extracellular matrix components. Transforming growth factor β (TGFβ) family members are the most important regulators in EMT [8]. TGF-β initiates and maintains EMT via Smad-dependent and Smad-independent signaling pathways [9, 10]. When TGFβ binds to its primary serine/threonine kinase receptor (type II), a heterotetrameric receptor complex is formed, then type II receptor *trans*-phosphorylates (activates) the signaling receptor (type I) that can initiate Smad2/3 signaling pathway. After ligand binding both TβRI and TβRII are rapidly internalized by clathrin-coated vesicles that target the complex into early endosome antigen-1 (EEA1) positive structures. These compartments promote the signaling providing platform for the downstream signaling elements to meet. Thus early endosomes (EE) play pivotal role not only in the sorting of internalized cargo proteins but in defining the activity of signaling events as well [11]. Another possible internalization route is caveolin-mediated endocytosis that targets TGF-βRs to lysosomal degradation. When the receptor—the signal transmitting structure—is removed from the cell membrane, the EMT signaling is stopped and the reverse process, the mesenchymal-to-epithelial transition (MET) can start.

In our experimental system we could show that the intraperitoneal injection of Freund’s adjuvant in rat induced a significant amount of TGF-β production in mesothelial cells that was secreted into the peritoneal cavity. The level of TGF-β highly correlated with the kinetics of the inflammation. Transforming mesothelial cells expressed TGF-βRII as well indicating that inflammation induced EMT in our system was also regulated by TGF-β signaling [12]. Our light and electron microscopic results showed that upon inflammatory stimuli TGF-βRII is internalized by caveolae, accumulated first in early endosomes, then detected in multivesicular bodies. These data provided evidence that caveola-mediated endocytosis assists in the attenuation of TGF-β signaling [12].

## Inflammation induced EMT transdifferentiates mesothelial cells into macrophages

We found that during inflammation-induced EMT mesenteric mesothelial cells start to express macrophage markers (OX43, ED1) as well [1, 13]This observation raised the possibility that mesothelial cells might transdifferentiate into macrophage-like cells [14]. This idea seemed to be even more likely since our previous data showed that under inflammatory stimuli the number of macrophages appearing in the peritoneal cavity dramatically increased [15]. Among these cells many were labeled by mesothelin (characteristic marker of mesothelial cells) and characterized by numerous caveolae at their plasma membrane [16]. Since inflammatory macrophages represent a heterogeneous cell population, the question arose whether these macrophage-like cells might contribute to this dramatic increase in number of macrophages during inflammation. Although tissue-resident macrophages, macrophages in the “milky spots” of the peritoneum, as well as infiltrating monocyte-derived macrophages play a distinct role in the progression of inflammation [17], we think, that these blood-derived monocytes and activated resident macrophages should not be sufficient to provide such a large number of macrophages appearing in the peritoneal cavity during inflammation. Cells originating from other, non-hematopoietic sources must contribute to the inflammatory subset of macrophages. Since mesothelial cells are present in huge numbers in the abdominal cavity covering the immense surface area of the intraperitoneal organs and mesentery, the idea, that these cells might transdifferentiate into macrophages, seems to be quite reasonable. This idea was further supported by our result, that mesothelial cells express nestin as well [1]. Nestin is a characteristic marker of multi-lineage progenitor cells, the presence of nestin indicates multipotentiality and large regenerative capacity [18]. Thus nestin expression in mesothelial cells strongly suggest that they are not terminally differentiated cells; instead, they represent multipotent “young” cell population with high regenerative capacity, and they are able to differentiate into many other types of cells [1].

We tried to find further evidences to support our idea that under inflammatory stimuli mesothelial cells can transdifferentiate into macrophages. Macrophages are professional phagocytic cells, they are the major producers of TNFα, which is the main regulator of inflammatory cytokine production [19]. Following the phagocytic activity of mesothelial cells we found, that upon inflammatory stimulus they efficiently started to phagocytose both India ink and fluorescent bioparticles [20] Control mesothelial cells do not express TNFα, but as the inflammation proceeded they expressed an increasing amount of TNFα as well [20]. EGR1 (early growth response 1) transcriptional factor is a member of early-immediate growth response gene family coupling the perturbation of the extracellular milieu to target gene expression [21]. As a unique transcription factor, EGR1 affects no other haematopoietic cells but the monocyte/macrophage lineage [22–25]. Control mesothelial cells do not produce EGR1, but inflammatory stimuli initiated the expression of this transcription factor, which was simultaneously translocated into the nucleus. EGR1 activity and its nuclear translocation depend on ERK activation [26]. Caveolin-1 is known to regulate ERK1/2 phosphorylation, positively affecting the monocyte to macrophage differentiation [26]. Following the time coarse of inflammation, we could detect an increased ERK1/2 phosphorylation, that reached the maximum level on 3^rd^ day. Parallel to this caveolin-1 expression has also changed, indicating that caveolin-1/ERK1/2/EGR1 signaling pathway plays crucial regulatory role in mesothelial cell-to-macrophage transdifferentiation [20].

Under inflammatory stimuli macrophages synthesize and secrete pro-inflammatory (TNFα, IL-1, IL-6, IL-8, IL-12), as well as anti-inflammatory cytokines (IL-10) [19]. Cytokines and chemokines are potent signaling molecules of low molecular weight, mediating intercellular communication. The primary function of cytokines is to regulate inflammation by paracrine, autocrine or endocrine mechanisms. Pro- and anti-inflammatory cytokines are mainly produced by macrophages and lymphocytes, but polymorphonuclear leukocytes, endothelial, epithelial cells, adipocytes and connective tissue cells are also able to synthesize them [19]. We found that control mesenteric mesothelial cells produce anti-inflammatory cytokine, IL-10, which is known to suppress macrophage activation, improves cell survival and reduces the level of inflammatory cytokines [27]. As the inflammation progressed, the IL-10 level produced by mesothelial cells decreased, they began to express the pro-inflammatory cytokine, IL-6 [28], regulating the expression of inflammatory genes [29]. At the peak of inflammation, IL-6 reached its maximum level, while IL-10 practically disappeared from the cells. As the regeneration started, IL-6 expression decreased and the cells started to produce IL-10 again [28].

## Internalization of receptor-ligand complex is essential for GM-CSF signaling to initiate mesothelial-to-macrophage transition

Since in an in vivo system the regulatory processes are highly complex, many factors are involved, we tried to choose one of these factors that can efficiently initiate EMT as well as the mesothelial cell-to macrophage transition. For this reason the granulocyte–macrophage colony-stimulating factor (GM-CSF) was chosen, which is a member of the hematopoietic cytokine family promoting the survival and activation of granulocytes, macrophages, and dendritic cell differentiation, in vivo*.* GM-CSF also stimulates proliferation of several non-hematopoietic cell types (osteoblasts, smooth muscle, endothelial and epithelial cells) [28]. When we treated mesothelial cells with GM-CSF, they lost their polarity, their contact with each other and with the underlying basal lamina. The cells became voluminous, and expressed macrophage markers [14], proving that GM-CSF itself had an inflammatory effect inducing EMT and mesothelial cells-to-macrophage transition. We also found that the mesothelial cells synthesized GM-CSF in vivo which was released (secreted) into the environment [14].

The biological effects of GM-CSF are mediated through binding to its cell surface receptor. The GM-CSF receptor is generally a heterodimer composed of a ligand-specific and ligand binding α (GM-CSFR α) and a signal transducer β subunit (GM-CSFR β), shared with IL-3 and IL-5 [30, 31]. Both receptor subunits are type I transmembrane glycoproteins [32], and their cytoplasmic domains are required for receptor complex activation [31]. Since the receptor does not have intrinsic tyrosine kinase activity, GM-CSFR β constitutively associates with tyrosine kinase JAK2. Binding of GM-CSF to its receptor leads to JAK2 autophosphorylation which then phosphorylates the signal transducers and activators of transcription 5, STAT5. As a dimer, the phosphorylated STAT5 migrates to the nucleus and binds specific DNA elements directing the transcription of genes related to cell survival, proliferation and differentiation [31, 32]. In our system we found that at the peak time of inflammation STAT5 was strongly phosphorylated on tyrosine residue, and detected in the nucleus of mesothelial cells as well, indicating that during mesothelial cell-to-macrophage transition STAT5 is activated, and the activated transcription factor translocated to the nucleus. We proved that mesenteric mesothelial cells express both GM-CSFRα and β receptors on their plasma membrane. During inflammation they synthesize and secrete GM-CSF that stimulates GM-CSF receptor expression as well as mesothelial cell-to-macrophage transition. Our results strongly suggest that GM-CSF, together with its receptor, are the major autocrine regulators in mesothelial cell-to-macrophage transdifferentiation.

We found that parallel to GM-CSF-induced EMT and increasing macrophage marker expression [14], the number of surface-connected vesicles (caveolae) dramatically decreased, suggesting that the GM-CSF signaling requires the internalization of receptor-ligand complex. Our double-label immunocytochemical studies showed that during GM-CSF-induced signaling, receptor β was internalized by caveolae. At the early time of inflammation, the receptor was delivered to early endosomes, where the signaling events of EMT (mesothelial-cell-to-macrophage transition) most probably started. We suppose that the activation of STAT5 occurs in early endosomes as well. Until the inflammation is maintained the receptor can be detected in recycling endosomes, indicating that the receptor can turn back to the cell surface, and taking part in another endocytic cycle the signaling is maintained [33]

To study whether internalization of the receptor/ligand complex is really necessary for the signaling, we used dynasore. Dynasore is a potent inhibitor of the small GTPase, dynamin, and efficiently inhibits the pinching off of vesicles from the plasma membrane [34], blocking both caveolin- and clathrin-mediated endocytosis. When the internalization of membrane-connected caveolae was blocked, GM-CSF treatment could not result in EMT, there was no ED1 expression and by blocking the receptor internalization the STAT5 signaling could not occur in mesothelial cells [14, 33].

These data provide strong evidence that the GM-CSF receptor internalization is indeed required to initiate signaling.

## Autophagy plays important role in regeneration, the mesenchymal-to-epithelial transition (MET)

After the peak time of inflammation the GM-CSFR β was present in late endosomes, proving that it has been transported to the degradative pathway. As the receptor degraded, EMT became gradually diminished and the regeneration (mesenchymal-epithelial transition, MET) started, the mesothelial cells gradually regained their simple squamous epithelial morphology and cellular organization [35]. The cells, being still cuboidal in shape, arranged in a single layer on the surface of the mesentery, the volume of the cytoplasm gradually decreased, and finally only few intracellular organelles were present in the cytoplasm [35]. Since no cell division was detected that could provide new mesothelial cells, we assumed that the regeneration of the mesothelium occurred by remodeling of the transformed mesothelial cells. This possibility seemed to be obvious because studying the time course of inflammation, parallel to the regeneration process progressive, autophagosome and autolysosome formation could be detected in mesothelial cells [35]. In tissue remodeling, autophagy is known to play a pivotal role, by which the cells can control the number and turnover of cell organelles [36–39]. Recent studies show that autophagy can play an essential role in inflammatory processes as well [40, 41]. In our system, we found that simultaneously with the morphological changes, the expression of various factors, directly or indirectly regulating autophagy, has also changed [35]. At the early time of inflammation the Beclin-1 level prominently increased, was high at the peak of inflammation, and by the time of regeneration completely disappeared. Beclin-1, member of the lipid kinase Vps34 core complex, is necessary for the formation of phagophores, thus it plays a crucial role in the induction of autophagy [42]. Although a basal Beclin-1 level is necessary for the cell survival, Beclin-1 alone is not capable to trigger autophagy [42]. The major regulator of the autophagy is mTOR, that integrates intra- and extracellular signals and serves a regulatory role in cell growth, proliferation, metabolism and survival [43]. Inhibition of mTOR increases autophagy, whereas its activation (by phosphorylation) reduces or inhibits the process [44]. mTOR is the downstream element of PI3K-Akt system. Various cytokines and extracellular stimuli can activate the serine/threonine protein kinase Akt or PKB (protein kinase B, a downstream effector of PI3K) as well as mTOR [45, 46].

The question arises what regulates, orchestrates the EMT, MET and autophagy in our system? It is well known that PI3K-Akt pathway can be activated by growth factors and cytokines [45, 47]. In our previous work we showed that during Freund’s adjuvant induced inflammation TGF-β is secreted into the peritoneal cavity [12, 48]. TGF-β is known to be the major regulator of EMT by activating the canonical Smad2/3 pathway, but it can also orchestrate many other signaling processes [49]. Besides inducing EMT, it can also activate indirectly the PI3K-Akt-mTOR pathway [50, 51] indicating that TGF-β has dual effect: through its plasma membrane receptor it stimulates EMT (canonical pathway) and at the same time indirectly inhibits autophagy (PI3K-Akt pathway). Since in our system TGF-β is secreted into the peritoneal cavity during inflammation [12, 48], we assume that TGF-β not only stimulates the EMT (through its canonical pathway), but it also arrests the autophagy by stimulating the phosphorylation of Akt-mTOR. As the inflammation progressed, and the autophagy was arrested, we found high p-Akt and p-mTOR levels [35]. Removing the TGF-β receptors from the plasma membrane by internalization, the TGF-β signaling was blocked, both p-Akt and p-mTOR levels diminished, the number of autophagic vacuoles increased significantly, indicating that when regulatory molecules were inactivated, the autophagy could be accelerated [35]. From these results is seems to be obvious that during inflammation TGF-β has indeed a dual effect in our system: it stimulates EMT and arrests autophagy. When we blocked autophagy by bafilomycin A1, the regeneration has not occurred, and the mesothelial cells died by apoptosis (unpublished data). These results further support the crucial role of autophagy in regeneration (MET). The receptor internalization, resulting in inactivation of PI3K-Akt-mTOR pathway, modifying the signaling events, can be one of the most important steps in accelerating autophagy.

## Conclusion

**Fig. 1:**
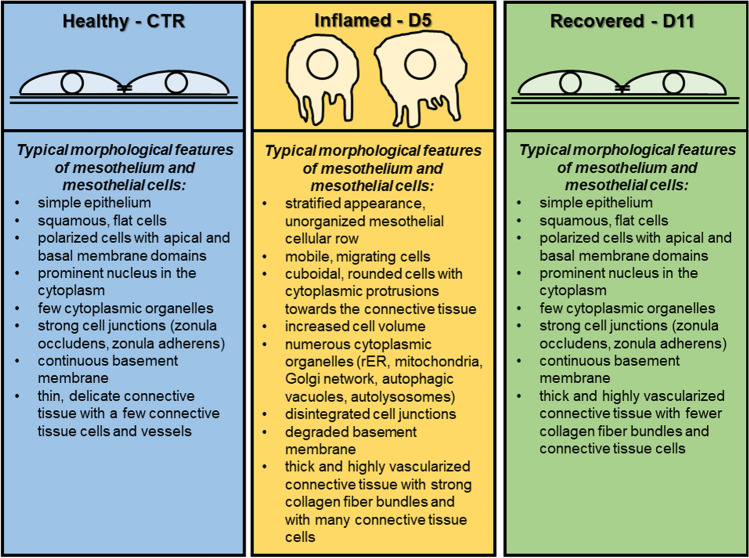
Morphological characteristics of control (healthy), inflamed and regenerated mesenteric mesothelial cells

**Fig. 2:**
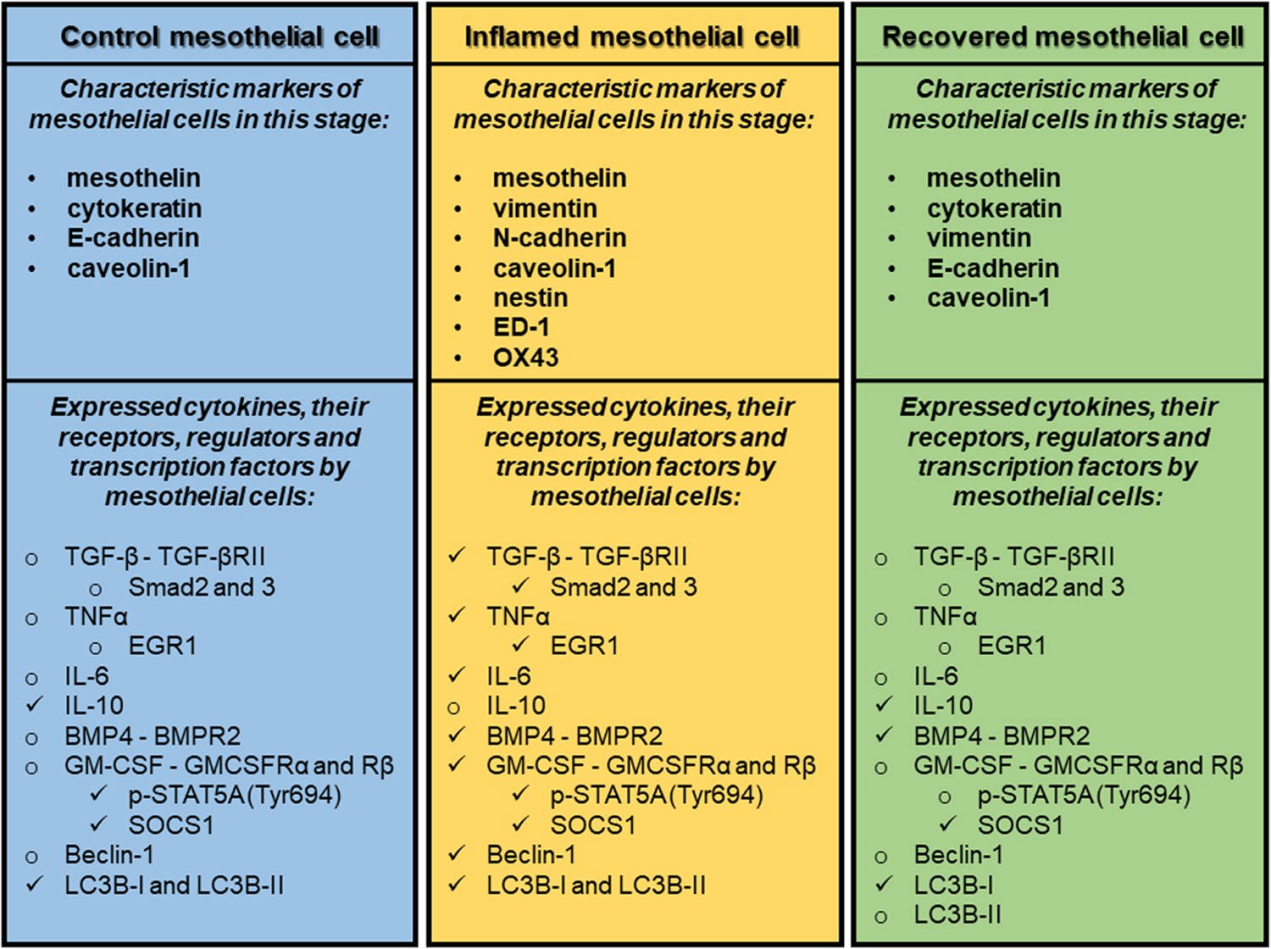
Molecular markers regulating EMT and MET during inflammation and regeneration of mesothelial cells in our experimental system. (Check mark labeled factors are expressed, factors labeled with empty circle are NOT expressed in mesothelial cells)

Our results provide additional evidences for the multipotency and plastic characters of mesothelial cells. Developing from the embryonic splanchnopleura, they preserve their multipotent mesenchymal character, they are not terminally differentiated cells, they can be taken as “stem” cells. In response to specific stimuli (inflammation, GM-CSF treatment) they can transdifferentiate into macrophages and in addition to emigrating blood monocytes and resident macrophages, these cells could provide the third source of peritoneal macrophages during inflammation. The internalization of cytokine receptor-ligand complexes is essential for the signaling of the mesothelial cell-to-macrophage transition. The EMT (inflammation, mesothelial cell-to-macrophage transition) is maintained until the receptor is recycling back to the cell surface. When the receptor is degraded, the regeneration (MET) starts indicating that EMT is reversible. Summarizing: during EMT mesothelial cells do not lose their mesothelial character, just because of being plastic, they are able to respond the environmental stimuli inducing epigenetic and cytoplasmic changes. When the inflammatory stimulus is over, and/or the surface sensors (receptors) are degraded, they can regain their mesothelial phenotype again (Fig. 1 and Fig. 2).

The future: Nowadays the Covid-19 pandemic events draw the scientist’s attention for our results. Our observations are especially important pointing out that the transformation of pleural mesothelial cells to inflammatory cytokine producing macrophages must play important role in the well-known cytokine storm, maintaining the inflammation and causing fibrotic alteration of lung alveoli. Thus the pleural mesothelial cells can be important pharmacological target.
